# A qualitative study investigating caregiver perspectives of an artificial intelligence assistive device to support daily activities in families with children with autism spectrum disorder

**DOI:** 10.1177/20552076251411228

**Published:** 2026-03-23

**Authors:** Nina Perry, Kelsie A Boulton, Lorna Hankin, Bruna B Roisenberg, Adam J Guastella

**Affiliations:** 1 Clinic for Autism and Neurodevelopmental (CAN) Research, Brain and Mind Centre, University of Sydney, Sydney, Australia.; 2 Children's Hospital Westmead Clinical School, Faculty of Medicine and Health, University of Sydney, Sydney, Australia.; 3 Child Neurodevelopment and Mental Health Team, Brain and Mind Centre, University of Sydney, Sydney, Australia.

**Keywords:** Adaptive functioning, artificial intelligence, AI, digital health support, qualitative

## Abstract

Children with autism spectrum disorder (ASD) and their families report diverse challenges in adaptive functioning. Artificial intelligence (AI)-assistive devices can offer innovative solutions to support adaptive functioning by offering real time assistance and capacity to adapt to heterogenous needs.

**Objective:**

This qualitative study utilised an AI-assistive device, the Pixi Home-Hub, to explore caregiver perspectives, experiences and needs related to the broader potential of AI to support adaptive functioning in their daily lives.

**Methods:**

Criterion purposive sampling recruited 10 caregivers of children with ASD. Two focus groups were conducted to discuss how an AI-assistive device, the Pixi Home-Hub, could support their child and families adaptive functioning. Content analysis was utilised to interpret the data. Independent coding by a second reviewer, analytical memos and peer review were employed to promote rigour.

**Results:**

Three key themes emerged from the data: (1) Caregiver experiences of their child's adaptive functioning in daily activities and areas for growth, (2) Caregiver experiences of balancing their child's support needs with their own, and (3) Access and barriers to integrating AI technology into everyday use.

**Conclusion:**

Caregivers believed that AI-technology can play a role in supporting their family's adaptive functioning activities and proposed using AI-assistive devices as a digital support navigator to facilitate greater access to health and government supports.

## Introduction

Autism spectrum disorder (ASD) is a neurodevelopmental condition (NDC) typically characterised by deficits in social communication skills and repetitive behaviours.^1^ Recent studies have revealed a growing prevalence of ASD,^2,3^ and it is estimated that 1 in 100 children will meet criteria for autism worldwide.^4^ This increase has been partially attributed to heightened global awareness and advocacy efforts.^5,6^ However, as demand for services and support has increased, it has become increasingly evident that many children and families affected by ASD share significant challenges in adaptive functioning. As a result, global organisations have increasingly drawn attention to the need for digital support strategies to assist both children and their caregivers in managing everyday tasks and participating fully in daily life. Moreover, there is growing recognition for assessments and supports that can cut across the diverse presentations of ASD in diagnostic settings,^7^ to break down service siloes that often exist (education, mental health and disability support needs) and to better address the diverse and complex support needs of people with ASD and their families.^8^

Adaptive functioning, now to be referred to simply as functioning, is defined as the skills necessary for an individual to navigate the demands of everyday life and to live independently.^9,10^ Functioning is a critical concept in assessing support needs for individuals with disabilities.^9^ In Australia, the National Disability Insurance Agency (NDIA) is the primary federal agency responsible for funding services for people with disabilities, with the objective to support functioning and enhance participation in everyday activities.^11^ In 2023, the NDIA cost the Australian Government nearly $30 billion, making it one of the country's largest expenses.^12^ However, approximately 84% of support in functioning activities has been found to be primarily provided by informal carers, such as parents and family members.^13^

Many caregivers report barriers to accessing care, with specialised services being in high demand with long waitlists.^14^ Families with autistic children often experience various challenges when it comes to navigating their child's functioning needs. These can include difficulty managing social, communication and learning needs,^15,16^ challenges with self-care activities, such as feeding, sleeping or dressing^17,18^ and obstacles when it comes to organising and managing competing responsibilities, such as work, school pick-ups, and the care of other children.^19^ Unfortunately, many of these challenges can be exacerbated by caregiver burden and stress, which has been found to be common in parents of children with complex needs,^20,21^ and often leads to diminished quality of life for both the child and the entire family.^15^ Concerns about the lack of access to specialist support and government funding,^14^ along with social stigma and isolation, place significant pressures on families.^22^ Further, high levels of financial stress associated with therapy, assessment, and school, as well as a lack of awareness from society of the challenges of having a child with autism, calls for a need for more supportive interventions to enhance quality of life and wellbeing for parents and their children.^22^ Given this, there is a pressing need to capture the experiences of caregivers to better understand how novel technologies can support their functioning needs in everyday situations.

These challenges in functioning serve as crucial benchmarks to address, and new technologies, such as artificial intelligence (AI)-assistive tools, may offer an innovative solution to target these. By using adaptive, data-driven approaches, AI-assistive technology may support the prioritisation of functioning and wellbeing needs of individuals with autism and their families by offering personalised support in real-time. AI is being used by people every day, but it has only recently emerged as a leading smart technology with the potential to improve health and wellbeing across medical disciplines.^23,24^ There has been increasing interest in utilising AI to improve the wellbeing of individuals with various health conditions and support needs,^25^ particularly as smart technology is increasingly utilised in homes.^26^ Due to the relatively recent developments in AI-assistive technologies, there is limited information on implementing these types of interventions, particularly for children and families living with autism. Previous studies have utilised social robots equipped with AI to initiate social communication behaviours in children with autism.^27,28^ Other studies involving children with autism have implemented interactive virtual reality systems and wearable devices to target social initiation behaviours.^29,30^ Griffen, Lorah^31^ and Bimbrahw, Boger and Mihailidis^32^ revealed increased daily living skills and independence in activities such as handwashing skills using tablet devices equipped with AI.^31,32^ Although the results emerging from these studies are promising, they should be interpreted with caution. A recent systematic review found that the majority of studies investigating AI technology to support functioning in NDCs have used small samples to test the feasibility of these technologies and have generally not included feedback from caregivers.^33^ In light of this, there has been increasing recognition that AI technologies need to be co-developed in collaboration with the community, to ensure that assistive devices are fit for purpose and provide maximum benefit to the child and family.^34^ By identifying and supporting adaptive functioning needs, there is the goal that AI technology can contribute to more streamlined delivery of services (e.g. eligibility, monitoring, and service delivery) and appropriate access to government funds.

This growing interest in AI creates new opportunities to align technological innovations with policy objectives aimed at improving the quality of life for people with disabilities. This has motivated multiple countries, including the United States,^35^ Denmark,^36^ Norway^37^ and the United Kingdom,^38^ to adopt frameworks that incorporate AI into their national disability strategies that aim to enhance accessibility and support for individuals with disabilities. In Australia, the NDIA delivers this structured support through the National Disability Insurance Scheme (NDIS). The NDIS provides a framework consisting of six core domains that aim to improve a person's functioning status, which includes mobility, communication, social interaction, self-management, learning and self-care.^39^ AI-assistive devices, such as apps, smartphones, tablets and interactive software, have been identified by the agency as a source of potential support, and major strides are being made to facilitate the growth of innovative assistive tools within future support schemes.^40–42^ This has culminated in the new NDIS framework to promote the development of AI-assistive devices, both to support a person's independence and help guide the development of new technologies.^43^ Notably, AI assistive technologies that currently exist do not comply with frameworks such as these as they do not possess the full range of functions that correspond to each of the framework's domains. Improving compliance between AI technologies and the NDIS framework could enhance funding access and break down barriers to support services, resulting in better outcomes for NDIS participants.^43,44^ Therefore, endorsement for assistive tools to align with these benchmarks is growing, with the goal that AI-assistive devices could serve a dual role by detecting functioning needs to more swiftly guide the delivery of supports provided by government services.

To harness these opportunities, qualitative feedback from the community is the first step to actively involve caregivers in designing technology that aligns with the NDIS framework for developing AI supports. This preliminary step in the co-design process allows for the design and implementation of AI-assistive devices to better meet the local needs of end-users whilst exploring potential issues that can be resolved before a device goes on the market.^34^ Furthermore, partnerships with industry ensures that AI-devices are developed and optimised in a rigorous manner and are aligned with real-world applications. The development of secure and high-performing AI technology relies on these collaborations but there is yet to be comprehensive research done in this area. In particular, there remains a gap in how researchers and industry can develop AI-assistive technology to meet the diverse needs of people living with autism. To ground this exploration, this study utilised a specific AI-assistive device, the Pixi Home-Hub, as a tangible probe to elicit caregiver perspectives, experiences and needs related to the broader potential of AI in their daily lives. We have chosen the Pixi Home-Hub specifically as it can provide support across all six areas of the NDIS functioning framework, although its impact is more substantial in areas of communication, social interaction, self-management and self-care, and comparably less pronounced in mobility and learning. For example, the calendar can be used to prompt planning of activities, goals can motivate self-care and the checklist can facilitate self-management. The device may also offer indirect support to less aligned domains through providing reminders for mobility-related exercises prescribed by therapists or help break down complex information into more manageable steps, assisting users to learn new skills or routines. The AI component of the Pixi Home-Hub provides predictive prompts in audio and visual formats to support the user's daily activities. These aspects of the Pixi Home-Hub can help caregivers by promoting consistency in routines and supporting functioning goals specifically for children with autism, thereby reducing caregiver stress and fostering greater independence in their children. Qualitative feedback offers a way to better understand the unmet needs that families may be experiencing, how technology equipped with AI can meet these needs, and what strategies, such as the NDIS, can be more effective through using these technologies. The collection of high-quality qualitative data is a crucial step towards successful integration of AI-assistive tools into everyday settings.

The aim of this study was to use focus groups to explore caregiver perspectives, experiences and needs related to the broader potential of AI to support adaptive functioning in their daily lives. In addition, this research aims to investigate the usability (e.g. ease of use, user satisfaction) of an AI-assistive device known as the Pixi Home-Hub to support functioning as reported by family members of children with autism. Partnering with industry to co-design a tool equipped with AI can also assist in better understanding how this kind of technology can create real positive impact for families. Through this collaboration, the future delivery of AI-assistive technologies can be better refined, and co-design engagement will better inform on the needs of the community.

## Methods

### Study design

This study was approved by The University of Sydney Human Research Ethics Committee (HREC 2022/AK0013) and conducted in accordance with the principles outlined in the Declaration of Helsinki.^45^ This study is a part of an on-going larger project, focusing on the feasibility of AI-assistive devices to support functioning in individuals with NDCs. This project is investigating an AI-assistive device that resembles an iPad known as the Pixi Home-Hub. The technology in the Pixi Home-Hub uses AI to adapt and respond to the user through an avatar known as ‘Pixi’. The Pixi Home-Hub also has functions embedded to improve areas of functioning, such as goals, to-do lists, tasks lists, contacts, a meal planner and a dashboard. This qualitative study adopted content analysis for data collection and analysis, as described by Elo and Kyngäs.^46^ The use of inductive content analysis was employed that allows for themes and patterns to emerge, and is useful to adopt when little is known about a topic.^46^ As caregiver experiences of their children's functioning in daily contexts and how an AI-assistive technology can be an effective support tool is an underresearched area, an exploratory qualitative study using content analysis was appropriate. Researchers employed open, nonleading questions during the focus groups. Additionally, analytical memos were taken to record the researcher's assumptions and beliefs about the topic during and after the study. Participants were familiar with the research clinic and selected through criterion purposive sampling via email invitation. Only caregivers participated in the focus groups, children did not use the device or take part in the study. As is typical in studies that utilise content analysis, a sample size of 5–10 individuals is necessary, but data collection continued until saturation was reached and no new themes or categories emerged from the data, ensuring depth was reached.^46,47^ The study was conducted at the Clinic for Autism and Neurodevelopmental (CAN) Research at the University of Sydney.

### Participants

Participants included caregivers who had attended either the Child Development Unit (CDU), a developmental assessment service located at The Children’s Hospital Westmead, or the CAN Research. Children presented to services for neurodevelopmental assessments or research studies. Eligible participants had a child aged between 2 and 16 years diagnosed with ASD as confirmed by their clinician. Informed written consent was gathered by researchers prior to participation in the focus groups. It was estimated that nine participants would yield a rich, in-depth understanding of caregiver experiences, and this was established as the provisional target.^48^ Ten caregivers (five female, five male) participated in the focus groups between July and September 2023. Two focus groups were conducted with four and six participants in each group. The group sizes helped foster in-depth discussions, increase participation and build rapport among participants. Focus groups were conducted in person and lasted on average 150 minutes.

### Industry Partnership

This study collaborated with a public benefit technology company (Akin Technology) located in Sydney, Australia, that develops AI-assistive technologies for people living with disabilities. This partner was involved in the provision of study materials for the focus groups (e.g. Pixi Home-Hub devices and tablet stands). The focus groups also included a representative from the industry partner to provide technology support. The industry partner was not involved in the analysis or interpretation of the study data.

### The Pixi Home-Hub device

**Figure 1.**
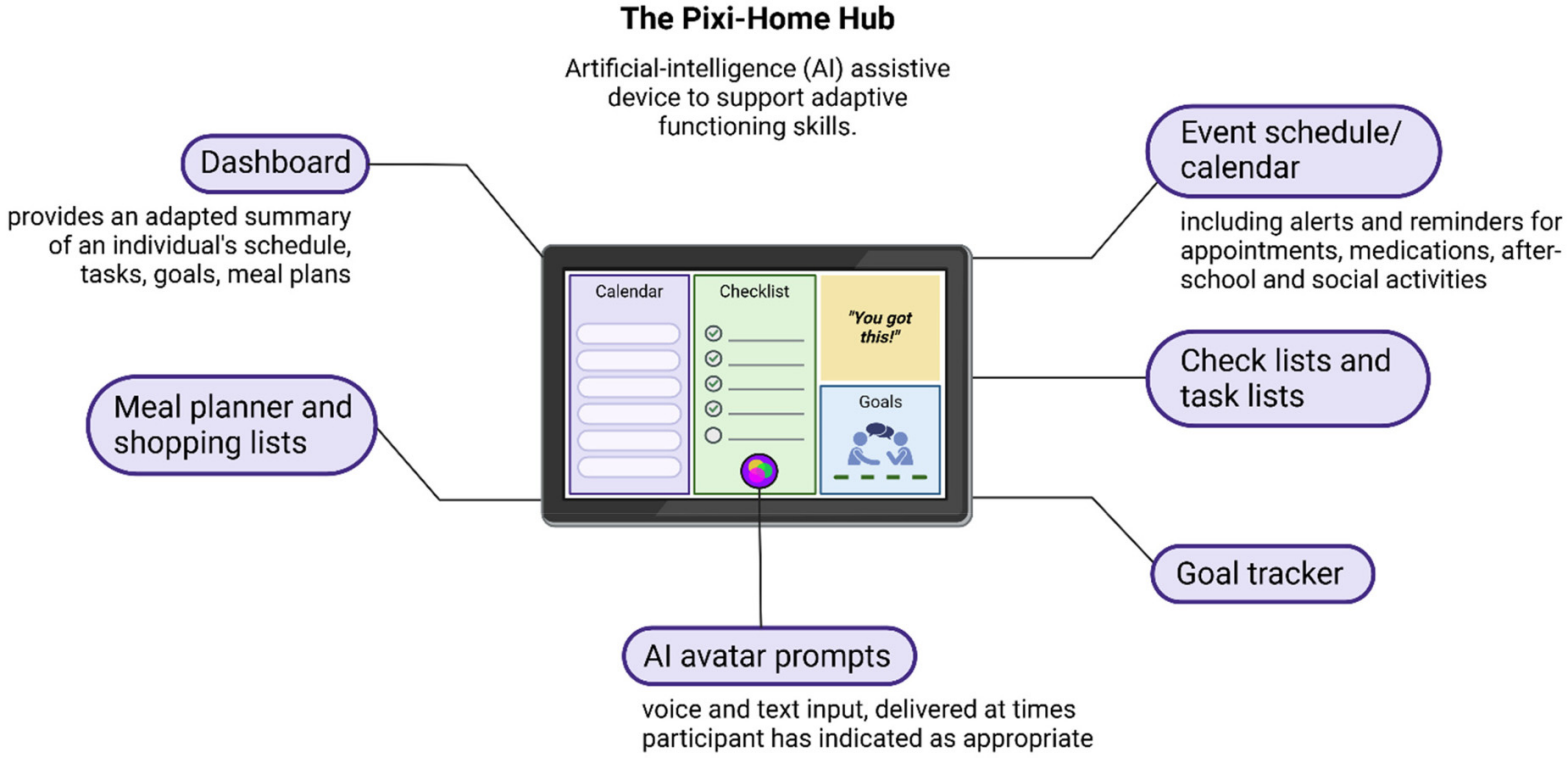
Overview of the Pixi Home-Hub device.

The Pixi Home-Hub device is programmed on a 10.5” (266.9 mm) Samsung Galaxy tablet A8 (see Supplemental Material). The programme is designed to provide adaptive assistance in daily living skills across different contexts (i.e. at home, school, workplaces), and serve as a central hub for managing daily tasks and functioning activities. The Pixi Home-Hub device was selected to use in this study for having multiple features, such as a calendar, meal planner, check lists and goals. These features can offer daily support to a broad range of individuals living with or supporting others with NDCs, which is a key outcome in this study. It is also currently approved by the NDIS and accessible to the public as assistive technology. AI-enabled prompts are delivered by the avatar Pixi, a voice controlled virtual agent that allows users to interact with the device using voice commands or text input. Pixi can answer questions, encourage engagement in social activities, provide information about tasks and upcoming events, as well as act as a general guide when navigating through the device. Other functions on the Pixi Home-Hub include an event schedule, calendar, check lists, task lists, goal tracker, and a meal planner (see Figure 1). All actions are summarised and presented on a dashboard. Users can interact with the Pixi device through text and audio inputs.

### Description of the focus groups

**Figure 2.**
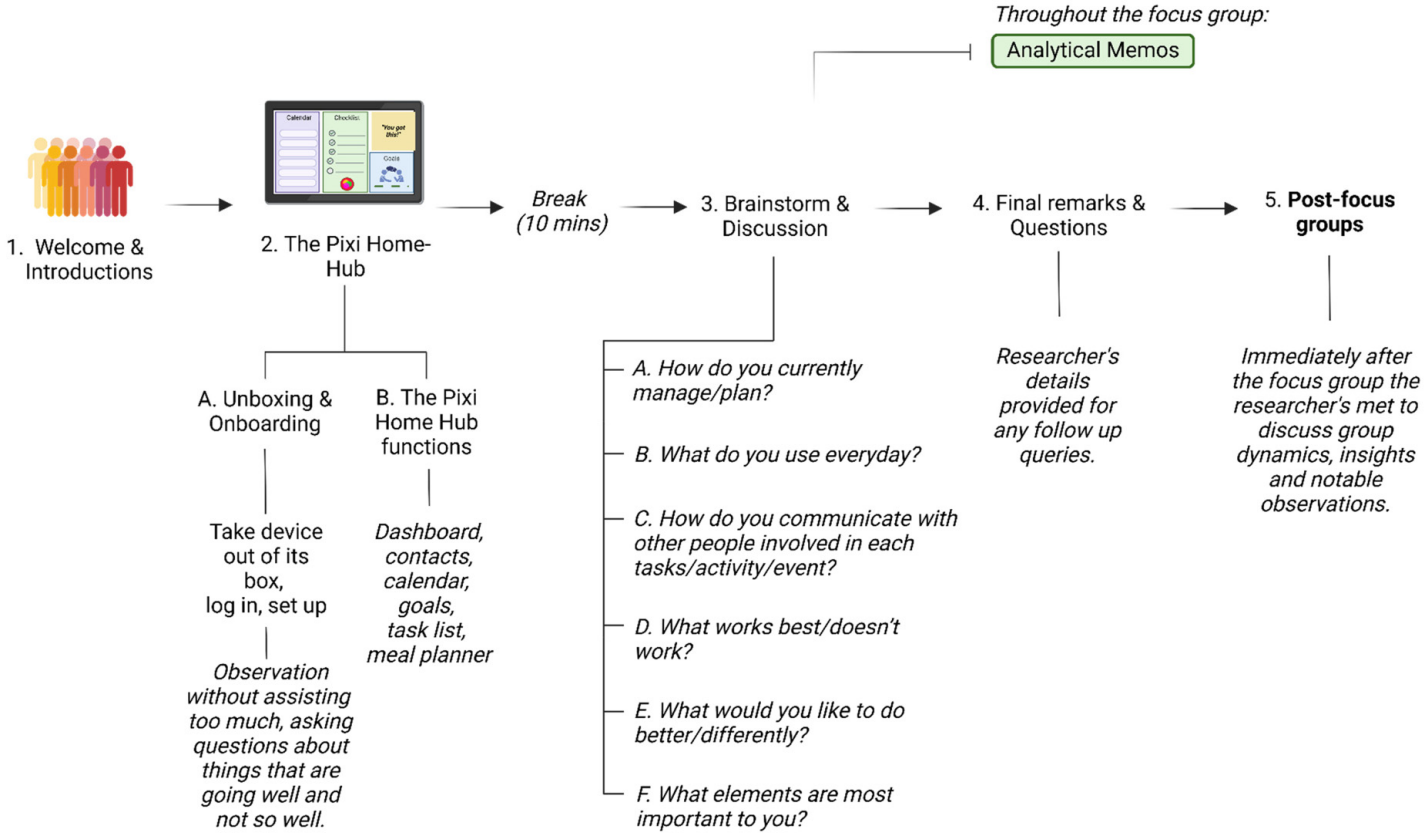
Structure of the focus groups.

Participants were asked to attend up to two focus groups to discuss aspects of managing their day-to-day life that are most important to them and what functions would be most useful to have in technology equipped with AI. Each focus group was capped at 5–10 participants to ensure each participant could share and contribute to the discussion; actual attendance resulted in one group of four and another group of six participants reflecting participants’ availability on the scheduled dates. Participants provided informed consent and were reminded to maintain confidentiality of what was shared by others. The focus groups were facilitated by two researchers (L.H. and N.P.) trained in qualitative methods. The structure of the focus groups was developed based on the research objectives and from consultation with our industry partner. The focus groups took approximately two and a half hours including a 10-minute break. These focus groups encouraged collaborative engagement to discuss the needs, interests, and motivations of using AI-assistive devices. During these focus groups, participants were tasked with exploring each feature of the Pixi Home-Hub device (i.e. the dashboard, task lists, goals, calendar, meal planner and interacting with the AI avatar ‘Pixi’) and providing feedback. The focus groups were structured with a brief introduction and overview of the Pixi Home-Hub, followed by an unboxing stage and open-ended questions regarding the device and participant's daily functioning activities (see Figure 2). Probing and follow-up questions were used to encourage deeper discussion and to explore emerging themes, with final remarks offering an opportunity for participants to summarise their thoughts and experience with the device. Analytical memos were taken by one facilitator (N.P.) to reflect on interactions with the device, as well as group dynamics and emerging themes. Facilitators held a debriefing session after the focus groups to discuss important observations. Both focus groups were video and audio recorded and transcribed by N.P. for analysis.

### Data analysis

The consolidated criteria for reporting qualitative research (COREQ) guidelines for qualitative research were followed.^49^ Content analysis was undertaken on full verbatim transcripts using NVivo v14, and transcripts were read until familiar.^50^ Analytical memos were taken to record ideas, interactions, and observations to aid in interpreting the data. This was done to improve credibility and enhance objectivity. Data collection and recruitment ceased once saturation occurred. Data saturation, as described in Guest, Bunce and Johnson,^51^ was defined as the point in data collection when no new themes emerged.^51^ Saturation was evaluated by two facilitators (L.H. and N.P.) after each focus group. After the first focus group, the facilitators reviewed emerging codes and themes and decided to proceed with an additional focus group. Following the second focus group, no new high-level themes were identified in comparison to the first focus group and data showed strong thematic convergence, suggesting data saturation was reached.

The subjective position of the researchers is displayed in Table 1. Intracoder reliability was achieved through familiarisation with the transcripts which occurred through multiple readings followed by coding of significant statements using an inductive approach. The data was re-read, re-coded and re-analysed at three time points to confirm saturation and collection of themes, as well as to ensure consistency in the interpretation and prevention of emerging biases.^52^ Identification of overarching themes that encapsulated commonalities between participants was undertaken independently and then refined further through revision and reflexive engagement with the dataset. Themes and illustrative excerpts are presented in a narrative table (see Supplemental Table S1).

**Table 1.** Description of researcher's subjective positions.

Theoretical framework	The theoretical framework used in this project was content analysis. This was primarily used to identify meaning and patterns regarding the daily lived experiences among families with children with autism and their experiences navigating an AI-assistive technology. An inductive approach to content analysis was employed, (no pre-existing categories and themes emerging directly from the data), which is useful when there is limited prior knowledge in an area of research.
Beliefs	Families with children with autism often exhibit challenges in functioning that can inhibit overall quality of life. Technology equipped with artificial intelligence can provide ways to support daily activities through tasks lists, goals, to-do lists and meal planners, which can be adapted to families’ specific needs.
Motivation	To explore the first-hand experiences of families with children with autism. To understand their point of view in matters most relevant to them in their daily lives, and to find better ways to improve support through AI technology. There is a lack of research on the experiences of families with children with autism and how they navigate functioning needs, further, there has been limited information on how AI can assist these needs. This qualitative perspective can aid in understanding these perspectives, which may be of interest to health professionals, researchers, and policymakers.
Researcher characteristics	Author N.P. is a female PhD student with postgraduate training in qualitative research methods and previous experience in neurodevelopment research with a focus on functioning and quality of life. She has no prior connections with the study participants. Author L.H. is a female researcher with postgraduate training in qualitative research methods and previous neurodevelopment research with a focus on clinical psychology and strengths-based approaches to child development. She has no prior connections with the study participants. Author B.R. is a female PhD student with postgraduate training in qualitative research methods and previous experience in neurodevelopment research with a focus on social functioning in people with autism. She has no prior connections with the study participants.

To obtain intercoder reliability, peer-review and debriefing with an independent second coder was employed. This method was chosen as most studies using content analysis theory do not incorporate intercoder reliability statistics and the study here aims is to explore caregivers experiences, not produce transferrable findings into clinical practice.^53,54^ The second coder (B.R.) was chosen as they had similar or equal experience in qualitative research as the first coder. The second coder (B.R.), blinded to the first coders initial interpretations of the data, independently coded 25% of the dataset, as it provides a substantial sample to assess consistency and reliability while still being manageable in terms of time and resources. Both coders (N.P. and B.R.) held team discussions regarding the unit of coding (randomly selected meaningful ‘chunks’), familiarisation with the coding scheme, coding exercises on NVivo software and established consensus procedures to clarify any ambiguities. Codes and themes were compared and discussed until consensus was reached. Of the 60 data segments coded from the seconder reviewer, the percentage disagreement was 18.3%. Disagreements (e.g. discrepancies in coding) primarily occurred in overlapping codes (i.e. ‘support needs’ and ‘NDIS’). All discrepancies were resolved through discussion among the coders of their interpretation and revisiting the data until a mutual understanding was reached, followed by refining the codebook. The coders reached agreement on 49 of 60 excerpts (81.7% agreement). Supervisors (A.G. and K.B.) were approached to review and provide independent assessments of any changes to the coding framework. To enhance trustworthiness of the analysis, reflexivity and rigour was maintained throughout by undertaking thorough revisions of the data, including codes and themes, as well as including the researcher's reflections and interpretations which were discussed with colleagues to identify additional perspectives. In addition, an audit trail was maintained to document changes in analytical decision making.

Open codes were generated by N.P. through inductive coding procedures. These were then grouped into 17 higher-order codes based on similarity, then refined into six subthemes that shared conceptual categories. Codes and sub-themes were generated through iterative refinement and thematic clustering, carried out over three separate revisions of the data. Subsequent analysis with a seconder coder (B.R.) led to the generation of three overarching themes that captured the central patterns across the dataset.

## Results

**Figure 3.**
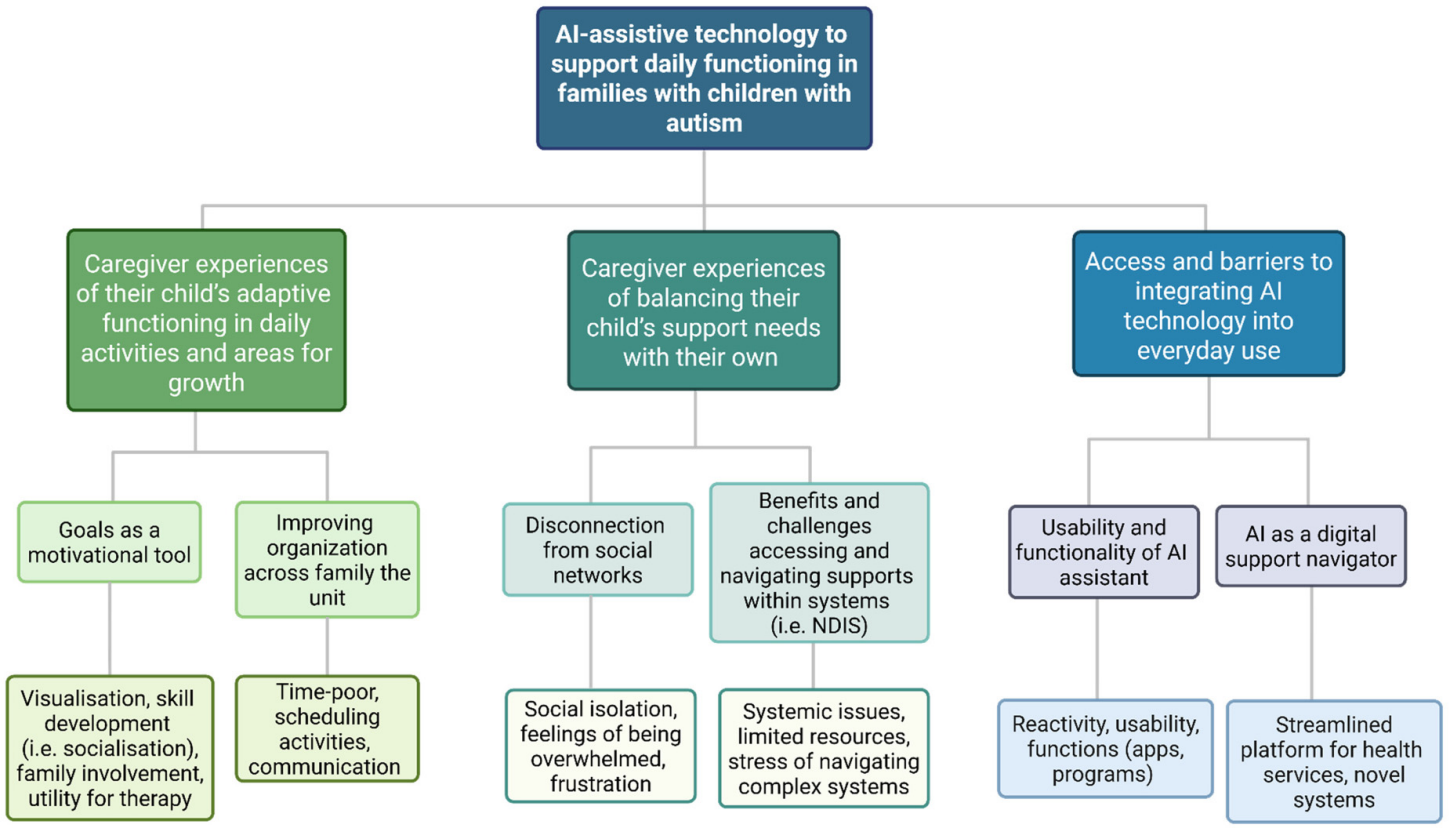
Coding Tree.

Abbreviations: AI: artificial intelligence; NDIS: National Disability Insurance Scheme.

Ten parents with children with ASD participated in the study, including five mothers and five fathers. The mean age of parents was 42.8 years and 80% spoke a language other than English at home (see Table 2). Children's ages ranged from 6.3 to 8.4 years and 90% were male. Children had received a diagnosis of ASD Level 2 (40%) and Level 3 (50%), defined as requiring substantial support in daily functioning. Three key themes emerged from the thematic analysis. While our focus was AI, two additional (non-AI) caregiver themes emerged. The three themes were: (1) Caregiver experiences of their child's adaptive functioning in daily activities and areas for growth; (2) Caregiver experiences of balancing their child's support needs with their own; and (3) Access and barriers of integrating AI technology into everyday use. Within these three themes, there were six sub-themes with corresponding codes, the description of the themes and sub-themes are presented in a coding tree (see Figure 3). Participants quotations and excerpts of the data are illustrated below, with additional examples presented in **Supplementary** Table S2. Subthemes are italicised and bolded.

**Table 2.** Demographic characteristics.

Participant	Parent age (years)	Parent education level	Occupation	Primary language spoken at home	Relationship	Child's age (years)	Child's gender	Child's ASD diagnosis	Child's secondary diagnosis (if any)
001	37.33	Postgraduate	NR	Nepali	Father	6.69	Male	ASD (Level 3)	Global developmental delay (moderate)
002	40.95	Tertiary	Self-employed	Vietnamese	Mother	7.15	Male	ASD (Level 2)	NA
003	50.57	Tertiary	Professional	English	Mother	7.12	Male	ASD (Level 3)	Global developmental delay (moderate)
004	NR	NR	NR	NR	Mother	NR	Male	NR	NR
005	35.71	Postgraduate	Professional	Punjabi	Father	6.49	Male	ASD (level 3)	Global developmental delay (moderate and speech impairment
006	47.13	Postgraduate	Professional	Telugu	Father	7.46	Female	ASD (Level 3)	Language delay
007	47.90	Postgraduate	Professional	Hindi	Father	6.30	Male	ASD (Level 2)	NA
008	39.08	Tertiary	Home carer	Hindi	Mother	6.30	Male	ASD (Level 2)	NA
009	36.86	Postgraduate	Self-employed	Mandarin and English	Mother	8.41	Male	ASD (Level 3)	NA
010	49.67	Postgraduate	NR	Bangali	Father	7.42	Male	ASD (Level 2)	NA

*Note:* ASD: Autism Spectrum Disord*e*r. NR: Not reported. NA: Not Applicable.

Definition of autism severity profiles: Level 2 (Moderate Support), Level 3 (Substantial Support). Specific data on socioeconomic status and educational attainment levels were not recorded. Participants 007 and 008 are parents of the same child.

### Caregiver experiences of their child's adaptive functioning in daily activities and areas for growth

The first theme was families’ experiences of their child's adaptive functioning in daily activities and areas for growth. This theme describes managing and adjusting daily activities and routines and the use of goals to facilitate greater independence, family involvement and therapy outcomes. Areas for growth refers to aspects where a child might need additional assistance, and areas of life families seek to improve on, such as organisation. Participants described using ***goals as a motivational tool*** to help their child complete tasks such as homework and therapy exercises. In addition, identifying goals on the Pixi Home-Hub was described positively as a productive and convenient way for caregivers to be involved in their child's healthcare journey. Participants could visualise using the Pixi avatar to learn more about their child's condition, skills and therapies in a way which communicates clinical information more simply and in a personalised manner. Being able to track data such as completed goals was also highlighted as motivational to have, due to the ability to see changes in their child's skills over time. Visualising developments this way was seen as helpful for many reasons, including being able to see their child's progress or challenges that were occurring in real-time and in different settings, as well as for tracking functioning behaviours in the long-term.

*These goals are set for the whole year, and we want them, but I want very small additions based on the skill set of my child. Why don’t we set this small goal? And I’ll be looking up, oh what's the next goal we can put, you know?* (P5_Father_35.7 y.o)

*Social or communication, language, fine motor skills or something like that and then I will track what's finished – like so for example, my son before is really focused more on social skills and now we focus more on like language skills. And then we can keep track of how he's going.* (P1_Father_37.3 y.o)

Caregivers also emphasised the importance of supporting social growth in daily activities and using assistive technology as a tool to better facilitate this. Streamlining activities such as playdates with other caregivers was considered important. For example, one caregiver noted using the Pixi Home-Hub to organise a playdate and it automatically being scheduled in their family's digital calendars with reminders. This was noted to help foster connections amongst other children and families in the community.

Secondly, using technology to ***improve organisation across the family*** unit was discussed as an important way to support children's functioning. Caregivers described their daily routines as being generally very busy. Caregivers reported that they would commonly use word-of-mouth or written reminders with their partners, or sometimes e-calendar invitations, to organise activities. Barriers would arise however when organising appointments or school-pickups with other relatives. Caregivers agreed that they were generally time-poor and more intuitive scheduling tools through AI could help improve communication and organisation amongst family members.

*For example, for my son. We might need to make a schedule for him. Like before school or like [when] he has piano or group therapy, [it] would be helpful. I really like it.* (P1_Father_37.3 y.o)

*So I think if you have a tablet like this, it would be really good if it could share calendars and things and you can put everyone in there to share that with people that are really involved in your lives.* (P3_Mother*_*50.6 y.o)

### Caregiver experiences of balancing their child's support needs with their own

The second theme was caregiver experiences of balancing their child's support needs with their own. This theme describes managing the demands and responsibilities of caring for a child that requires significant support, while also attending to their own needs and wellbeing. Caregivers reported adjustment to their social lives and activities, as well as their reliance on support systems (i.e. family, professional services) to help manage these responsibilities. Utilising disability support resources, such as the NDIS, was a crucial part of balancing care and everyday activities.

Firstly, caregivers discussed being ***disconnected from social networks***, including a lack of social participation, feelings of isolation and frustration. Caregivers expressed that AI technology could support their needs, by automatically scheduling appointments, setting reminders, and prompting engagement in social activities. Furthermore, when asked whether they experienced social isolation, caregivers across both focus groups responded yes, highlighting that this was a shared experience amongst caregivers.

*I get disconnect[ed] with my friends because I keep on forwarding many of the events. When I tried to put [them] in my phone, after a few days I forget. Nowadays, like how we are living, if they understand us, they are our friends, otherwise let's just move on. Now some things feel like disconnected.* (P2_Mother_40.9 y.o)

Secondly, parents experienced ***benefits and challenges accessing and navigating supports within systems*** (e.g. NDIS in Australia). Participants reflected that although accessing supports was a frustrating and emotionally draining process, it did assist in supporting their child's needs (e.g. using speech therapy services for socialisation skills). This subtheme contributes to the broader theme of challenges accessing and navigating supports because it captures the sentiment that although support was beneficial, getting to that point was not always straightforward and sometimes difficult to engage with. This tension highlights that perceived value of support does not eliminate the barriers to access it. It was also apparent that caregivers wanted the process of accessing their NDIS supports to be simpler and less time consuming. Most challenges stemmed from systemic issues with government support, such as underfunding and eligibility barriers.

*He was about to achieve all of his goals, he had around 60–70% he had achieved and then the plan was reviewed, and they cut the [NDIS] funding to 20%. 20% is nothing. I’m also working full time, how much more money do we need to pay how much everything do we need to pay? I need to reduce my working hours, everything I need to reduce.* (P8_Mother_39.1 y.o)

Other challenges mentioned were more context dependent, such as limited resources to access certain types of assistance. For example, changing the type of specialist supports. In addition, families reported the stress of navigating complex support systems and emphasised the desire for more streamlined supports.

*Where it falls down is when you shift your therapies. For example, my son he doesn’t need speech [therapy] so much, but he might need social therapies. It's really hard to edit your NDIS report with those changes and then all your schedules change as well. So you’re taking out speech [therapy], you’re adding in social therapy and then suddenly everything's a bit crazy.* (P3_Mother_50.6 y.o)

*You know, I feel that this software and NDIS can give us access to a huge amount of money in lots of proper ways, so we can give proof to the NDIS and say look, focus on the client don’t focus on the therapist.* (P10_Father_49.7 y.o)

### Access and barriers to integrating AI technology into everyday use

The third theme was access and barriers to integrating AI technology into everyday use. This theme describes aspects of adopting AI technologies, like the Pixi Home-Hub, into people's homes and daily activities. Feedback on Pixi's functionality and usability were discussed, as was how the Pixi Home-Hub can be optimised as a digital support navigator for functioning, a use of AI devices caregivers were enthusiastic about pursuing.

Firstly, there were some concerns surrounding the ***usability and functioning *** of Pixi**,** which was viewed as slow or unresponsive at times. This made it hard to interact with the AI component. It was observed by the facilitators and documented in analytical memos, that this factor made people lose interest interacting with the AI avatar and resulted in caregivers moving on to interact with the other features of the device during the focus groups.

*Because when we talk to an AI device straight-away we say, “Hey Google, I need to set an alarm” and we don’t wait for it to be ready, this thing's got to be ready – I’ve never heard a word back from Pixi so far.* (P5_Father_35.7 y.o)

Integrating AI features, such as personalisation and automatic responses, on the task lists, to-do lists and goal functions were met with interest and caregivers could see themselves using these in daily settings to support their child's functioning, such as in training social skills.

*So if one of the parents say, you know “[child 1] wants to have a playdate with [child 2] next Saturday, how about it?”, you can put it in and it just sends you the info automatically.* (P3_Mother_50.6 y.o)

Secondly, other aspects of the Pixi Home-Hub such as the meal planner, were seen as helpful as an idea but not in practice due to restricted or routine eating patterns that caregivers experienced with their children. Furthermore, most participants described current access to technology in their everyday lives. They did not see it particularly necessary to incorporate an additional device and would prefer a system like Pixi to be an app or service that could be accessed on devices they owned already.

*Because as a device it becomes a lot to maintain and to keep with me, so I think it would be more appropriate as an app. On like your phone where it can integrate your calendar and my activities.* (P3_Mother_50.6 y.o)

Caregivers provided ideas for the future development of the Pixi Home-Hub, primarily by using ***AI as a digital support navigator*** in their daily lives. Participants suggested that the Pixi Home-Hub would be most useful to them in navigating healthcare systems by integrating different service providers. As a digital support tool, caregivers proposed the idea of the Pixi Home-Hub streamlining service and funding information onto one digital platform to automatically track and update support plans (i.e. for the NDIS) to best meet the functioning needs of their children.

*I think it would be really helpful with NDIS planning. So you have your calendar. You have your documents, you have all these things, and you have your NDIS portal. Everything sits everywhere. It would be really good if you could find a particular way for all of these to talk to each other.* (P3_Mother_50.6 y.o)

There was active discussion about the ways in which the device could be a useful tool to connect various people involved in their child's care with the NDIS outcomes framework. In this sense, it was suggested that a network of allied health professionals, clinicians and teachers could communicate with one another using the Pixi Home-Hub to improve the sharing of information regarding their child's care. Bridging all sectors of the families’ healthcare system together with their funding plans on a single device was suggested to improve the experiences of caregivers navigating supports. Eliminating the need to keep hard copies of information was desired, and storing reports and therapy information digitally in one place would facilitate better sharing of information with clinicians. Furthermore, tracking finances between medical services with funding for supports was recommended as an avenue for further development.

*Yeah, you know I’ve got 2 [children]! So, on top of the invoices, you know, you not only got to pay them. You’ve got to keep them. They are for the NDIS. So, the invoices and the reports’ part I think in terms of what I do, takes up 80% of my time.* (P3_Mother_50.6 y.o)

Caregivers identified that this would support their time and resource management, planning, and task prioritisation by having the ability to track information in a central place rather than through multiple formats (i.e. printed reports, emails, notes, electronic records). Having this streamlined into one platform was mentioned as a novel approach to assist in their daily lives and would be beneficial when re-applying for government funding.

*Everything, having the plan provider, the NDIS should be the main channel then I believe it [the Pixi Home Hub] will help us to proper utilise funding and show what we’re doing every day.* (P10_Father_49.7 y.o)

## Discussion

This study sought to explore the diverse experiences, perspectives and needs of caregivers of children with ASD as they engaged with AI technology. Caregivers reported that they would prefer to use an AI-assistive device as a digital support tool to help them navigate their child's health and functioning needs. Participants wanted technology with intuitive information gathering and personalisation features, designed to monitor their child's functioning and development. Functioning activities were desired to be tracked primarily through goals that can be designed by families and the child's healthcare teams, supplemented with personalised suggestions from AI. Caregivers agreed that this information would be useful when planning funding supports, particularly through federal support agencies (e.g. NDIS), which was an area of life that was revealed to be time-consuming and burdensome. Streamlining data-driven approaches that captures their child's functioning across different settings (i.e. at home, school), with their clinician's reports that are then linked to funding requirements, was considered by caregivers as a novel approach to using an AI-assistive device like the Pixi Home-Hub.

This study adds to the growing research on the use of AI technology to support children with autism. In comparison to previous research, which has largely investigated robotics^27,28^ virtual reality systems and wearable devices,^29,30^ this study addresses a critical gap by exploring the use of an AI-assistive tablet device with direct feedback from end-users. In addition, compared to previous studies utilising AI to support single functioning skills (i.e. socialisation),^55^ this article reports on a technology that can aid in a broad range of daily functioning activities that are directly linked to current policy requirements for support. Much of the feedback caregivers provided coincides with established frameworks to support functioning skill development in children with autism. These include setting activities as goals and reinforcing positive achievements (i.e. Applied Behaviour Analysis)^56,57^ and practising skills through instructions and prompts (i.e. Social Skills Training, Task Analysis).^58,59^ AI-assistive tools offer a new way to translate these frameworks into practice. As revealed by Bandura,^60^ goal attainment is essential for encouraging behaviour and self-efficacy (i.e. the belief in one's capabilities to accomplish tasks), which can help formulate independence.^60^ In children with ASD, fostering self-efficacy is crucial for several reasons. Executive skills are frequently delayed in these children,^61^ but by promoting self-efficacy through goal setting, there may be significant improvements in executive function, behavioural outcomes, academic performance, and social confidence.^62–64^ Furthermore, assisting children develop a strong sense of their abilities through accomplishing goals can go on to support development across broader domains of functioning, such as independence in daily living skills and relationships.^65,66^ In this study, caregivers could see this supported by the Pixi Home Hub by presenting suggestions of goals that were generated intuitively by AI and their child's clinicians, and subsequently scheduled automatically into their child's routine. This application is yet to be seen in research, however, a review by Rehman et al.,^67^ recommends that future applications of AI for people with ASD should include similar features such as those in the Pixi Home-Hub, including progress tracking and personalisation.^67^

Caregivers of children with ASD are becoming more recognised in research, and it has been established that caregiver functioning is an important predictor of functioning behaviours in their children.^68,69^ Caregivers in this study reflected on how they face barriers when it comes to daily life, such as maintaining social relationships, managing competing tasks and staying organised. These experiences are echoed by McAuliffe et al.,^70^ who found that mothers of children with autism play a significant role in organising and managing family routines, and this was often at the cost of their own health and well-being.^70^ Although this article emphasises the mother's role, in this study we found that fathers reiterated similar experiences in managing daily life. In addition, social isolation experienced by parents of children with ASD is well-documented and is known to lead to negative health outcomes,^71–73^ this is a significant concern and an area that requires intervention and attention. Resources aimed at facilitating greater social wellbeing for parents have been found to operate as a protective factor against negative health outcomes.^74^ Interestingly, evidence suggests that functioning support through technology can be a successful way to foster greater connection to the community amongst families and children with disabilities.^75^ Conversely, there still remains a shortage of research exploring how technology can assist parents with daily life, especially in managing everyday tasks and social activities, since most studies have primarily concentrated on individuals with neurodevelopmental diagnoses. Understanding caregiver experiences reinforces the need to prioritise caregiver perspectives, including developing more caregiver-centric frameworks in neurodevelopmental research.

In this study, parents reported that the value of support does not negate the struggle to access it. Support systems, such as the NDIS, need to be more accessible so parents can better engage with it. A review by Veli-Gold et al,^76^ investigating families experiences navigating the NDIS indicated that carers find it difficult to access information about the NDIS and the planning process is emotionally burdensome.^76^ Caregivers in this study shared similar experiences, particularly when it came to coordinating and communicating with the NDIS. Parents of children with disabilities often find navigating support services challenging due to a number of factors, including a lack of information about the services available to their child,^77^ issues coordinating between different service providers,^78^ and the emotional stress of feeling like they have to constantly advocate for their child's needs.^79^ A major barrier caregivers discussed in this study was financial barriers to accessing the appropriate supports. This was described by some caregivers as funding for certain therapies and activities being taken away when their child is still benefiting from them, resulting in a decline of skills. Previous research has documented similar barriers experienced by caregivers,^14^ such as the unpredictable or high cost of specialised services.^80–82^ Caregivers provided multiple suggestions on how AI-assistive devices could help them overcome these barriers. For example, capturing functioning progress or needs in areas of daily life to show where financial assistance can be better allocated, as well as streamlining and storing financial documentation such as invoices, receipts, and upcoming payments in one place to ease the process of making NDIS reports. This reiterates that the experiences of caregivers navigating supports can be oftentimes challenging, indicating a need for governments and organisations to work towards creating more effective and accessible support solutions for families.

### Implications for AI-driven digital support navigation tools

The results of this study have translatable implications for families, clinical services, policy and practice. Research applying different AI features such as speech-to-text software, conversational AI and personalised reminders in people with different disabilities have found improvements in functioning^83–86^ and caregivers in this study could envisage the future utility of the Pixi Home-Hub once some functionality problems were resolved (i.e. Pixi being relatively slow or unresponsive to voice prompts).

#### Connecting a network of care providers

Research investigating digital support navigator tools is gaining attention, particularly in areas such as mental health, reflecting growing momentum towards ways we can leverage technology to enhance assistance and support for people with complex needs.^87–90^ The application of AI into these tools has not yet been explored. In the current study, caregivers reiterated the importance of developing intuitive technology to connect their child's functional development with healthcare teams, teachers, and other family members.

#### Streamlining supports and funding

Linking a child's developmental information with support services, such as the NDIS, was suggested to improve families’ experiences of navigating multiple services for their children. Networked and connected care practices across organisations, such as hospitals and governments, are anticipated to take place over the next 10 years.^24^ AI embedded into digital supports may offer significant benefits for precision medicine across these domains, enabling more efficient use of health services and funding. For example, by automating assessments, adjusting care plans and analysing service use data.^24,91,92^ In this scenario, AI support navigation tools could provide quick, tailored interventions and minimise delays in funding approvals so families can access support sooner.

#### Integrating service providers

Greater personalisation of support and faster service delivery is essential for the functional success of families with children with autism.^93–95^ Harnessing AI technology can help facilitate the shift from static to needs-based service models. For example, to automatically adjust financial support based on the caregiving environment or matching individuals to community programmes based on their unique social and health profiles.^91,96^ Using digital support navigation tools to streamline this can benefit both families and the wider healthcare system by directing resources where they are most needed. In this sense, the Pixi Home-Hub could act as a nexus between healthcare providers, educational support, government assistance providers, and other caregivers, to form collaborative links among their child's wider community. This kind of streamlined digital navigator is yet to be reported on and the caregivers in this study have helped present this novel solution to support functioning skills for their families.

### Strength and limitations

The strengths of this study include the sample characteristics, comprised equally of mothers and fathers of young children with autism with diverse cultural and linguistic backgrounds. The flexibility of data collection through focus groups allowed for new topics to be explored as they arose naturally, thereby allowing diverse viewpoints to be gathered and detailed insight into the lived experiences of caregivers with autistic children which is beyond what traditional quantitative measures can capture. This study also employed strategies to encourage rigour such as peer review of the coding methodology with verification by an experienced researcher (A.G., K.B.). This study explores a focused research question in a specific participant group, which further supports saturation of data. This study is not without its limitations. Although our analysis produced rich insight into caregivers’ experiences, these findings are not intended to be generalisable. First, the AI system was slow to respond, which could have affected data completeness. Additionally, participants did not comment on issues related to ‘trust’ in the AI system (i.e. data privacy and ethical considerations). This lack of discussion represents a limitation in capturing an important area of awareness and potential concern as AI continues to evolve. Furthermore, potential selection bias may have influenced the study's results, the sample consisting of parents with children primarily on the severe range of the autism spectrum and very early adopters of this technology, limits the generalisability of the results to broader populations. Finally, the research team acknowledges its own reflexivity as a methodological consideration and recognises that their perspectives may have influenced data interpretation. Overall, focus groups were an effective method for exploring complex issues and generating ideas. The focus groups used in this study have been useful in informing further research and practical recommendations for future refinement of the Pixi Home-Hub device. Finally, while we acknowledge that funding was provided by the industry partner (Akin Technology) to conduct an evaluation, they had no role in the data collection, analysis or writeup of this manuscript. There is an urgent need for evidence-based practices to drive technology developed by industry. The intention of this evaluation was to conduct an initial evaluation to begin evidence collection.

### Future research

Future research in this area might consider conducting additional focus groups with children and caregivers, as well as seeking guidance from caregivers following the integration of these changes and ascertaining whether an optimised device is better at meeting their functioning needs. In addition, the application of the Pixi Home-Hub, once optimised, in at-home trials would be the ultimate goal and is a part of the broader scope of this research. This would allow caregivers to experience the usability of the device in their daily routines and offers researchers an opportunity to gather experience-sampling data to detect whether functioning can be improved upon through AI technologies. The results of this study cannot be extrapolated to all families with a child with an autism diagnosis, however, they will help optimise the interoperability of a device such as this to address issues experienced by caregivers. We anticipate that the findings of this research will add value to future iterations of the Pixi Home-Hub.

## Conclusion

The conclusions drawn from this study indicate that families with children with autism would benefit the most from AI assistive technology that can support goals and social skill development, as well as improve organisation across the family unit. This study also found that caregivers experience their own challenges with functioning, including social disconnection, which is an area that requires further attention. These findings indicate that the most utility an AI-assistive device would have for families is the ability for it to be a single digital support navigator to organise and navigate supports, allowing for data-driven evidence of their child's functional development to be communicated across their health networks. The information gathered from this research advocates for collaborating with industry and end-users to develop an AI-assistive device and suggests that, if designed to meet diverse needs, integrating AI into caregivers’ daily routines could benefit families. Future research should explore the use of AI assistive technologies in different settings to assess their impact across different environments and people.

## Supplemental Material

sj-docx-1-dhj-10.1177_20552076251411228 - Supplemental material for A qualitative study investigating caregiver perspectives of an artificial intelligence assistive device to support daily activities in families with children with autism spectrum disorder Supplemental material, sj-docx-1-dhj-10.1177_20552076251411228 for A qualitative study investigating caregiver perspectives of an artificial intelligence assistive device to support daily activities in families with children with autism spectrum disorder by Nina Perry, Kelsie A Boulton, Lorna Hankin, Bruna B Roisenberg and Adam J Guastella in DIGITAL HEALTH
