# Supplementary material for: A qualitative study investigating caregiver perspectives of an artificial intelligence assistive device to support daily activities in families with children with autism spectrum disorder
Source: Digit Health. 2026 Mar 23;12:20552076251411228. doi: 10.1177/20552076251411228 (PMC13009858; doi:10.1177/20552076251411228)
Supplement: sj-docx-1-dhj-10.1177_20552076251411228 - Supplemental material for A qualitative study investigating caregiver perspectives of an artificial intelligence assistive device to support daily activities in families with children with autism spectrum disorder [file sj-docx-1-dhj-10.1177_20552076251411228.docx]

## **Supplementary Figure 1: Example Screenshot of The Pixi Home-Hub Interface**

**
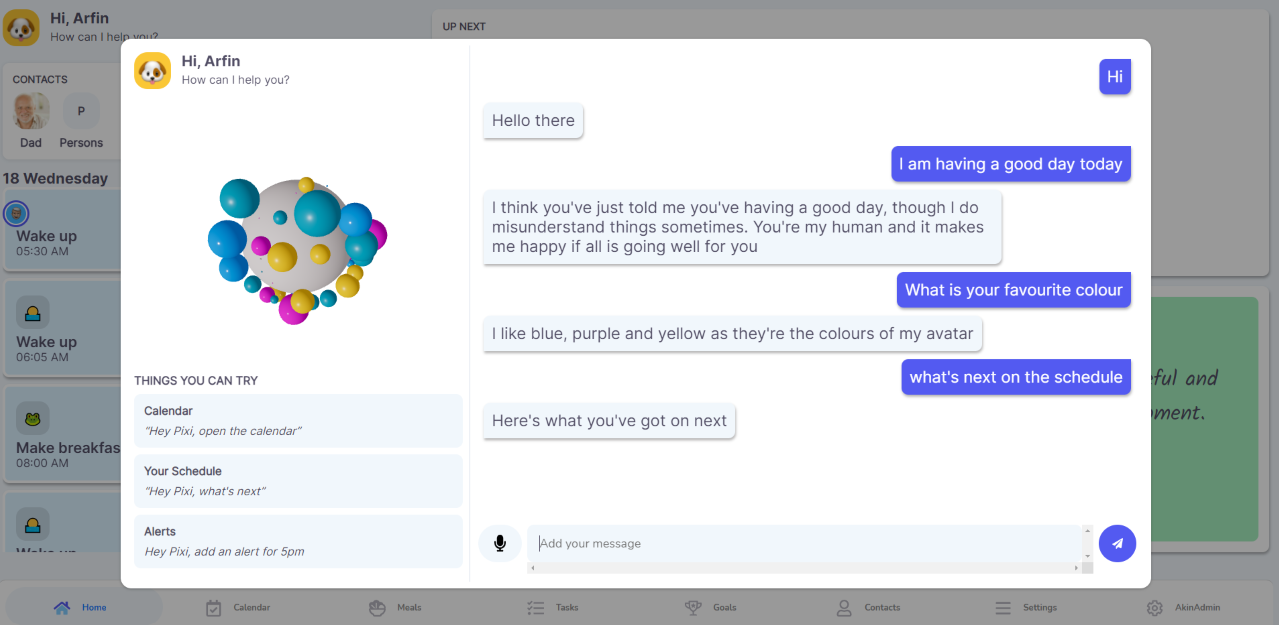
**

# **Supplementary Table 1: Narrative Table of Themes, Subthemes and Codes**

| **Themes** | **Subthemes** | **Codes** |
| --- | --- | --- |
| Caregiver experiences of their child’s adaptive functioning in daily activities and areas for growth | Goals as a motivational tool | Visualisation, skill development (i.e. socialisation), family involvement, utility for therapy |
|  | Improving organization across family the unit | Time-poor, scheduling activities, communication |
| Caregiver experiences of balancing their child’s support needs with their own | Disconnection from social networks | Social isolation, feelings of being overwhelmed, frustration |
|  | Challenges accessing and navigating supports  within systems (i.e. NDIS) | Systemic issues, limited resources, stress of navigating complex systems |
| Access and barriers of AI technology into everyday use | Usability and functionality of AI assistant | Reactivity, usability, functions (apps, programs) |
|  | AI as a digital support navigator | Streamlined platform for health services, novel systems |

# **Supplementary Table 2: Additional Quotations from Focus Groups**

| **Themes** | **Example Quotes** |
| --- | --- |
| Caregiver experiences of their child’s adaptive functioning in daily activities and areas for growth | *“I really like the goal part, like at the top, where it's got things you could, like suggestions. Like is it eating healthy, exercise. I really like that. I think that's really good.”* – 003  *“Like oh, social or communication, language, fine motor skills or something like that and then I will track whats finished – like so for example my son before is really focused more on social skill and now we focus more on like language skill and maybe like 2, 3 goals doing maybe half a year? And then we keep track of how he’s going”* – 001  *“I guess its visual, right, guys? I think we do, as parents, we as a parent I think sometimes, you know, like my son's speech was terrible now he can talk to anyone. You can tell at home too how they’re sort of tracking, like how they’re going. Sometimes it goes forward, sometimes it goes back.”* - 003  *“Like for example, it gives me the option to set a goal but as a parent with a son who has ASD, I didn’t even know what ASD stands for it’s a big challenge for parents to know what their goals should be for their children.”* – 005  *“Because as a device it becomes a lot to maintain and keep it with me, so I think it would be more appropriate as an app. On like your phone where it can integrate your calendar and my activities and and and somehow if its able to integrate with my therapists, NDIS, and school, it would be more efficient.”* – 005 |
| Caregiver experiences of balancing their child’s support needs with their own | *“I don’t know how people do it without help. I know some people do do without help, I don’t know how. We have a cleaner that comes once a week. My Mum helps with washing clothes. If not, I’d just be like drowning.”* *–* 003  *“It’s like there’s always. There is always something in our mind like about the kids and things like that, about their future we always like get lost thinking about their futures and things like that. So like, we are always lost. Actually.”* – 002  *“It’s really the people who have got it in their home, if somebody doesn’t have an autistic kid or something, they will not understand people like us. Other people who doesn’t know about it, they don’t understand it. In past, you would go to the playground and we’d have to tell them this thing is happening. Yeah, this is this thing. So it can be really hard.”* – 002  *“Same thing I asked for funds for my son’s support worker, they refused to give because they asked me who is living with you, I said my brother and my Mum. Our Mum can follow him around the house, up and down, up and down, but that’s not respectful you know? And my brother, they refused to give me money for a support worker because they said you have your brother to support you. Why? My brother wants go outside to earn money, why would he stay here and look after my son?”* – 008 |
| Access and barriers of AI technology into everyday use | *“First of all I know these devices have come so far, people expect it to be very fast, responsive, very responsive, whereas this app is very slow. When you click you have to think about whether you clicked or not and then you realise that you’ve clicked and people are not very patient”* – 005  *“All I can really say is that I don’t see it as a device. Because as a device it becomes a lot to maintain and keep it with me, so I think it would be more appropriate as an app. On like your phone where it can integrate your calendar and my activities and and and somehow if its able to integrate with my therapists, NDIS, and school, it would be more efficient.”* – 005  “*Visual is really important because, you don’t know but we are always in a trial and error. Something comes out after some time and if you can capture that in some way, its good but its very difficult. So here you have a video and you can pass it on to show he was actually doing it this way, then they can continue and they can properly take care of him.”* – 010 |

## **Supplementary Figure 2:** **Consolidated criteria for reporting qualitative research (COREQ): a 32-item checklist for interviews and focus groups**


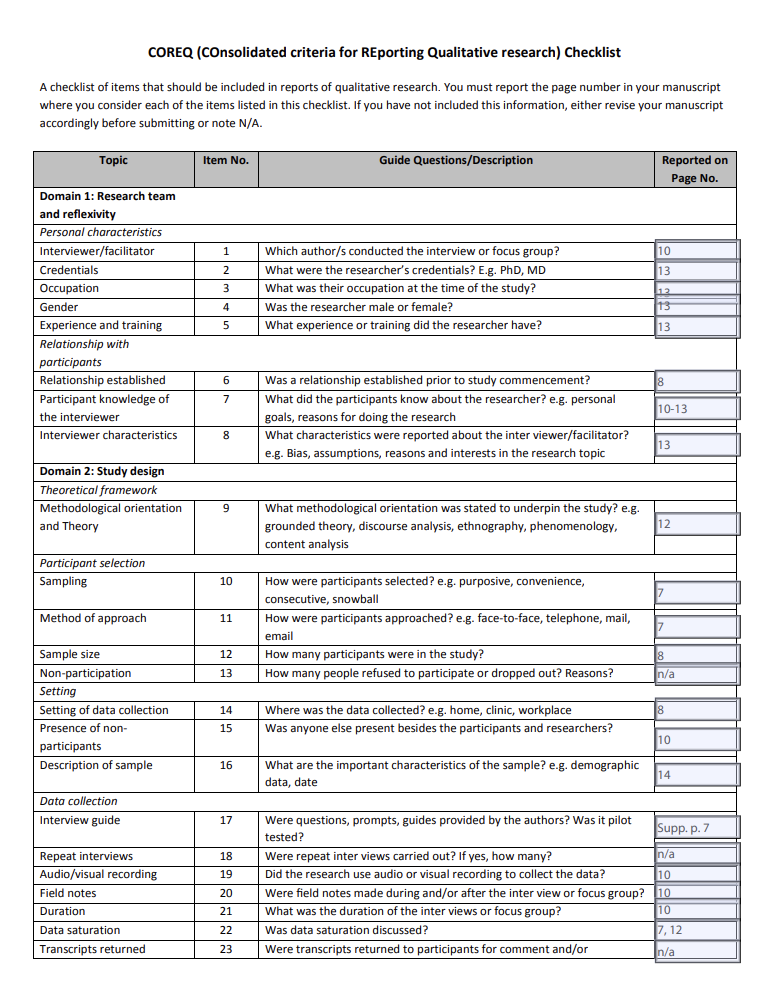


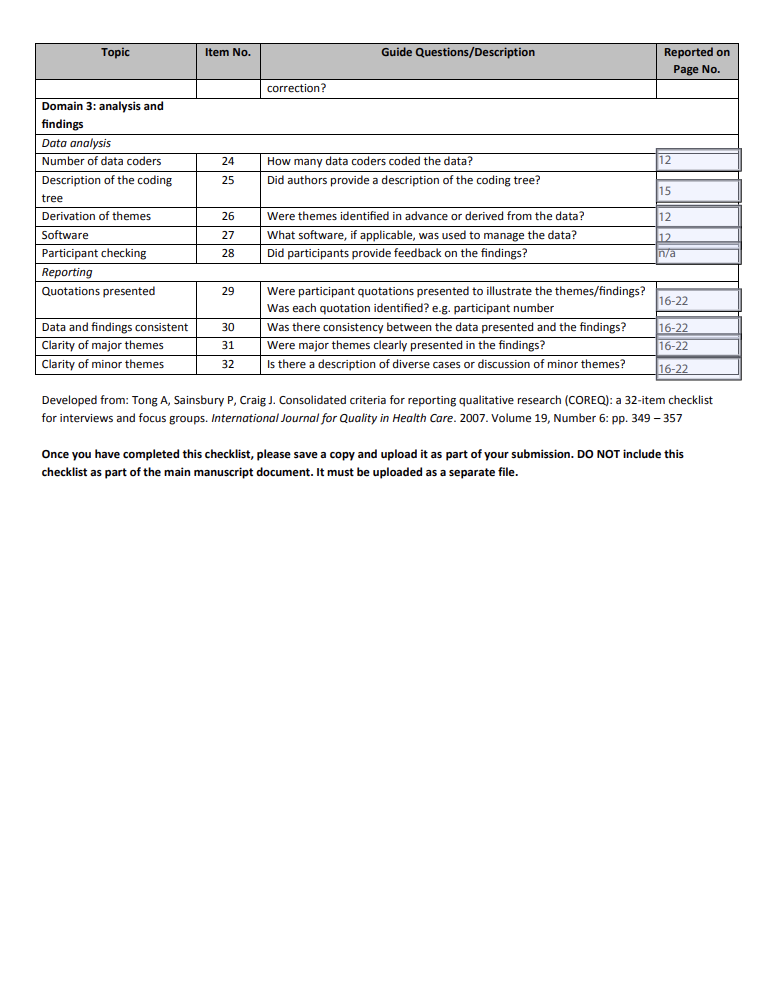


**Supplementary Table 3:** **Focus Groups Interview Guide**

| **Type** | **Question/s** |
| --- | --- |
| 1. Welcome & Introduction | [*Participants are introduced to facilitators* [*omitted for peer review*] *and a representative from akin*] |
| 1. Introducing the Pixi-Home Hub device | [*Background information on akin (public benefit company who developed the Pixi-Home Hub) and research project is explained*] |
| 1. Unboxing and Onboarding Session | [*Participants take the Pixi-Home Hub out of its box, log in and set up its functions independently. Researchers* [*omitted for peer review*] *observing*]  Questions:   1. How are you finding logging in so far? 2. Are you finding this easy/hard? |
| 1. Break | 10-minutes |
| 1. Brainstorm | Questions:   1. How do you currently manage/plan:  - Scheduling appointments and activities? - Tracking your child’s therapy and goals? - Daily/weekly tasks, such as shopping, cooking, cleaning, childcare? - Juggling work/home-life?  1. What do you use? (Apps/software/notebooks/folders/post-its…) 2. How do you communicate with other people involved in each task/activity/event? 3. What works/doesn’t work? 4. What would you like to do better/differently? 5. What elements are most important to you? 6. What’s important in Pixi? 7. What did you like? 8. What could be better? 9. What else would you like it to do? |

**Supplementary Table 4: Frequency of codes**

| **Theme** | **Number of Participants**  **(n = 10)** |
| --- | --- |
| Caregiver experiences of their child’s adaptive functioning in daily activities and areas for growth | 39 |
| Caregiver experiences of balancing their child’s support needs with their own | 32 |
| Access and barriers of AI technology into everyday use | 9 |

*Note: frequency does not indicate equal importance but supports transparency of the study findings.*
